# A New Deep Learning Calibration Method Enhances Genome-Based Prediction of Continuous Crop Traits

**DOI:** 10.3389/fgene.2021.798840

**Published:** 2021-12-17

**Authors:** Osval A. Montesinos-López, Abelardo Montesinos-López, Brandon A. Mosqueda-González, Alison R. Bentley, Morten Lillemo, Rajeev K. Varshney, José Crossa

**Affiliations:** ^1^ Facultad de Telemática, Universidad de Colima, Colima, Mexico; ^2^ Centro Universitario de Ciencias Exactas e Ingenierías (CUCEI), Universidad de Guadalajara, Guadalajara, Mexico; ^3^ Centro de Investigación en Computación (CIC), Instituto Politécnico Nacional (IPN), Esq. Miguel Othón de Mendizábal, Mexico city, Mexico; ^4^ International Maize and Wheat Improvement Center (CIMMYT), Texcoco, Mexico; ^5^ Department of Plant Sciences, Norwegian University of Life Sciences, IHA/CIGENE, As, Norway; ^6^ Centre of Excellence in Genomics and Systems Biology, International Crops Research Institute for the Semi-Arid Tropics (ICRISAT), Hyderabad, India; ^7^ State Agricultural Biotechnology Centre, Centre for Crop and Food Innovation, Murdoch University, Perth, WA, Australia; ^8^ Colegio de Postgraduados, Montecillo, Mexico

**Keywords:** genomic selection, genomic prediction, calibration of predictions, deep learning, GBLUP, plant breeding

## Abstract

Genomic selection (GS) has the potential to revolutionize predictive plant breeding. A reference population is phenotyped and genotyped to train a statistical model that is used to perform genome-enabled predictions of new individuals that were only genotyped. In this vein, deep neural networks, are a type of machine learning model and have been widely adopted for use in GS studies, as they are not parametric methods, making them more adept at capturing nonlinear patterns. However, the training process for deep neural networks is very challenging due to the numerous hyper-parameters that need to be tuned, especially when imperfect tuning can result in biased predictions. In this paper we propose a simple method for calibrating (adjusting) the prediction of continuous response variables resulting from deep learning applications. We evaluated the proposed deep learning calibration method (DL_M2) using four crop breeding data sets and its performance was compared with the standard deep learning method (DL_M1), as well as the standard genomic Best Linear Unbiased Predictor (GBLUP). While the GBLUP was the most accurate model overall, the proposed deep learning calibration method (DL_M2) helped increase the genome-enabled prediction performance in all data sets when compared with the traditional DL method (DL_M1). Taken together, we provide evidence for extending the use of the proposed calibration method to evaluate its potential and consistency for predicting performance in the context of GS applied to plant breeding.

## Introduction

Genomic selection (GS) exploits dense genome-wide markers for predicting complex traits. Practically it requires development of a training population (with phenotypic and genotypic information) with which a statistical machine learning algorithm is trained and used for making predictions for individuals of a test breeding population with only genotypic information. Genome-enabled prediction and GS were originally proposed by Meuwissen et al. (2001) as a novel approach for predicting complex traits for a selection of candidates using predicted phenotypic or breeding values. Zhong et al. (2009) and Heffner et al. (2010) state that GS works given realistic assumptions of selection accuracies, breeding cycle times and selection intensities. In simple terms, GS offers tremendous opportunities to improve rates of genetic gain in plant and animal breeding, and it has been supported by many research articles published in the last 20 years (Bhat et al., 2016; Crossa et al., 2017).

GS is changing the landscape of practical plant breeding, as it is able to predict breeding values earlier and with greater accuracy when compared with conventional selection methods such as mixed models, Ridge regression and Bayesian methods (BayesA, BayesB, BayesC, Bayesian Lasso, etc). Additionally, time is saved by using GS because it is no longer necessary to wait for late filial generations to phenotype complex quantitative traits such as yield, biotic and abiotic stresses, among others. The genotypic data can be obtained from the seed of early generations and used to predict phenotypic performance of later generation individuals without the need for extensive phenotyping evaluation over years and environments (Mellers et al., 2020). Furthermore, it highlights the potential to increase the speed of varietal development across crop species (Bhat et al., 2016; Crossa et al., 2017).

Estimating the genetic worth of the individual in GS is based on a large set of marker information distributed across the whole genome, which contrasts with the relatively few markers used in marker assisted selection (MAS) (Varshney R. K. et al., 2021). Conventional breeding involves hybridization between diverse parents and subsequent selection over a number of generations to develop improved crop varieties. This has several limitations, including the long duration (5–12 years) required to develop a crop variety, the reliance on time-consuming (and traditionally low-throughput) phenotypic selection, high environmental noise and genotype × environment interactions. It is also less effective for complex and low heritability traits (Tuberosa, 2012). For these reasons, several studies have shown GS models to be advantageous for complex quantitative traits like grain yield, quality, biotic and abiotic stresses, etc. (de los Campos et al., 2009; Crossa et al., 2010; Burgueño et al., 2012; González-Camacho et al., 2012; Jannink et al., 2010).

However, there are still numerous opportunities to improve the selection process of candidate individuals in GS. Some of these are: 1) to improve the quality and coverage of marker data; 2) to design optimal training-testing sets; 3) to better identify where in the breeding program GS could be efficiently applied (Crossa et al., 2017); 4) to have sufficient numbers of individuals in the reference (training) population; and 5) to use the most appropriate statistical machine learning model for each data set at hand.

Intensive research has explored different statistical machine learning methods for GS (Varshney R. K. et al., 2021). For example, some of the models/methods used in GS are: 1) linear mixed models and their Bayesian counterpart that includes the so-called Bayesian alphabet [BayesA, BayesB, BayesC, Genomic Best Linear Unbiased Predictor (GBLUP), and Bayesian Lasso]; 2) Random forest for predicting binary, categorical and continuous traits (Montesinos-López et al., 2021a); 3) support vector machine (Montesinos-López et al., 2019a); 4) gradient boosting machine and 5) deep learning algorithms (Montesinos-López A. et al., 2018; Montesinos-López, 2018b; Montesinos-López et al., 2019a; Montesinos-López et al., 2021c). These statistical machine learning methods have been adopted for GS because they can help improve genome-enabled prediction accuracy as they use machine learning advances for analysis, interpretation, prediction and decision-making. One explanation of why many statistical machine learning methods have been implemented in GS is the fact that there is no universal best prediction model that can be used under all circumstances (No free lunch theorem; Wolpert, 1996).

Deep learning (DL) methods are one of the most recent adoptions of statistical machine learning methods used for GS (Varshney RK. et al., 2021). There is mounting evidence suggesting that these methods outperform conventional methods in terms of predictive power, as well as other advantages (Montesinos-López et al., 2021c). Some of these advantages are: 1) power in capturing complex patterns in the data caused by the inclusion of many neurons communicated in complex ways and via multiple nonlinear transformations through hidden layers (Montesinos-López et al., 2019b; Montesinos-López et al., 2021c); 2) support for raw (not preprocessed) inputs, which is impossible with most statistical machine-learning methods (Montesinos-López et al., 2021c); 3) support for a variety of different inputs that can accommodate pedigree, genomic, environmental and other forms of omics data (e.g., metabolomics, microbiomics, phenomics, proteomics, transcriptomics, etc.) (Montesinos-López et al., 2021c); 4) greater efficiency for handling large and complex data sets compared with most statistical machine-learning methods (Montesinos-López et al., 2021a,c); and 5) a very flexible network architecture permitting a “Lego-like” construction of new models, while an unlimited number of neural network models can be constructed using elements of the core architectural building blocks of existing DL models (Montesinos-López et al., 2021a, c).

While DL methods offer many advantages, their training process is very challenging, especially considering hyper-parameter selection. The correct selection of hyper-parameters are time consuming and complicated to implement largely due to the absence of a unique and efficient optimized methodology. This means that the implementation of DL methods for genome-enabled prediction is not straightforward. Furthermore, DL methods are inefficient when used with small data sets or with simple linear patterns, and as such, research is underway to facilitate the training process of DL methods so that they can be used in these contexts.

One way of improving the training process of DL models is to use a calibration based on a model that is already trained and applied via a post-processing operation. However, in the context of machine learning methods (including DL), calibration methods have only been proposed for binary and categorical response variables, where the predicted probabilities that do not match the expected distribution of the observed probabilities of the response variable in the data are adjusted (calibrated) to increase this match and the prediction accuracy in the testing set. This is very important in classification problems because the estimated class probabilities reflect the true underlying probability of the sample. For this reason, the predicted class needs to be well-calibrated, which means the probabilities must effectively reflect the true likelihood of the event of interest. This discrepancy between the distribution of observed and predicted values is also very commonly found in continuous response variables, and yet no solution has been proposed. In other words, this means that despite all efforts taken during the training process of the deep neural network, often times, there will still be bias in the predictions. Consequently, methods for calibrating continuous and categorical response variables are of paramount importance to increase the accuracy of your prediction machine.

Based on the previous considerations, the main objective of this study is to present a method that facilitates the calibration of DL outputs in the context of genomic-based prediction in GS. In this vein, we propose a calibration method for continuous response variables that significantly improves the training process for DL methods. We used four existing data sets to compare the prediction accuracy in terms of Mean Squared Error Prediction (MSE) of the popular Genomic Best Linear Unbiased Predictor (GBLUP), for the standard DL method (DL_M1) and the new proposed calibration method (DL_M2). GBLUP was used for comparison, as it is the most used model in genome-enabled prediction. The genome-enabled prediction models and methods were also compared in the absence and presence of genotype × environment interaction.

## Materials and Methods

Data sets used in previous studies were employed here for assessing the performance of the new calibration methods applied to DL.

### Dataset 1. Maize Grain Yield Prediction

As previously reported by Montesinos-López et al., 2016 this dataset consists of a sample of 309 maize lines evaluated for three traits: anthesis-silking interval, plant height and grain yield (GY). Each trait was evaluated in three optimal environments (denoted Env1, Env2 and Env3). It is important to point out that each line was evaluated once in each environment, and as such, each lines has three replications. Additionally, it should be highlighted that we have genotypic information for the 309 lines and phenotypic information for the 927 (309×3) observations, which were all collected in the same year. The field design in each of the three environments was a lattice incomplete block design with two replications. Data were pre-adjusted using estimates of block and environmental effects derived from a linear model that accounted for the incomplete block design within environments and for environmental effects. The lines were genotyped with 681,257 single nucleotide polymorphisms (SNPs). Markers with more than 20% missing values and with minimum allele frequency (MAF) of 0.05 were removed. The remaining missing markers were imputed using observed allelic frequencies resulting in 158,281 SNPs available for further analyses. In the present study, we compared genome-enabled prediction performance for GY.

### Dataset 2. Groundnut Seed Yield per Plant (SYPP) Prediction

The phenotypic dataset reported by Pandey et al. (2020) contains information on the phenotypic performance for various traits in four environments. In the present study we assessed predictions using the trait seed yield per plant (SYPP) for 318 lines in four environments denotes as Environment1 (ENV1): Aliyarnagar_Rainy 2015; Environment2 (ENV2):Jalgoan_Rainy 2015; Environment3 (ENV3):ICRISAT_Rainy 2015; Environment4 (ENV4):ICRISAT Post-Rainy 2015.

The dataset is balanced, giving a total of 1272(318×4) assessments (phenotypic values) with each line included once in each environment (four replications of each line), which were all measured in the same year. Marker data were available for all lines and 8,268 SNP markers remained after quality control (each marker was coded with 0, 1 and 2); however, the makers were obtained only for the 318 lines.

### Dataset 3. Chickpea Biomass Prediction

The phenotypic dataset reported by Roorkiwal et al. (2018) contains information for 315 lines evaluated in six environments (denoted as 1, 2, 4, 5, 6, 7) for biomass. The dataset is balanced with all lines assessed in all environments (that is, four replications of each line), giving a complete phenotypic dataset with 315×4=1890 observations. Marker data were available for all 315 lines, with 35,527 SNP markers available following quality control, where each marker was coded with 0, 1 and 2, and all information was collected in the same year.

### Dataset 4. Spring Wheat Grain Yield Prediction

Spring wheat data was available from the Global Wheat Program (GWP) at the International Maize and Wheat Improvement Center (CIMMYT) from elite yield trials (EYT) evaluated in four selection environments (denoted Bed5IR, EHT, Flat5IR, FlatDrip). The dataset included the performance data from the 2016-2017 cycle from a total of 980 lines assessed in the four environments, giving 3920(980×4) observations since each line was repeated four times, once in each environment. The experimental design was an alpha-lattice with the lines sown in 39 sets, each including 28 lines and two checks in six blocks with three replications. Four performance traits were assessed:days to heading (number of days from germination to 50% spike emergence); days to maturity (number of days from germination to 50% physiological maturity or the loss of green color in 50% of the spikes); plant height (measured from the ground to the top of the spike, in centimeters); and grain yield (GY). Genome-wide SNP markers were generated for the 980 lines using genotyping-by-sequencing (GBS; Elshire et al., 2011; Poland et al., 2012) at Kansas State University using an Illumina HiSeq2500. After filtering, 2,038 markers remained. Imputation of missing marker data was done using LinkImpute (Money et al., 2015) implemented in TASSEL V5 (Bradbury et al., 2007). In the current study we assessed predictions using GY.

### GBLUP Model

The model assumed for the response variable was

$$Y_{ij}=\mu +Loc_{i}+g_{j}+gL_{ij}+\varepsilon_{ij}$$ (1.1)

where $Loc_{i}$ are the fixed effects of locations, $g_{j},$ j=1,…,J , are the random effects of lines, $gL_{ij}$ are the random effects of location-line interaction, and $\varepsilon_{ij}$ are random error components assumed to be independent normal random variables with mean 0 and variance $\sigma^{2}$ . Furthermore, it is assumed that $g={\left(g_{1},\ldots ,g_{J}\right)}^{T}∼N_{J}\left(0,\sigma_{g}^{2}G\right)$ , $gL={\left(gL_{11},\ldots ,gL_{1J},\ldots ,\;gL_{IJ}\right)}^{T}∼N_{IJ}\left(0,\sigma_{gL}^{2}\left(I⊗G\right)\right)$ **,** where G is the genomic relationship-matrix as computed by VanRaden (2008) and ⊗ denotes the Kronecker product. The implementation of this model was done in the BGLR library of Pérez and de los Campos, 2014.

### Conventional Deep Learning (DL_M1)

**FIGURE 1:**
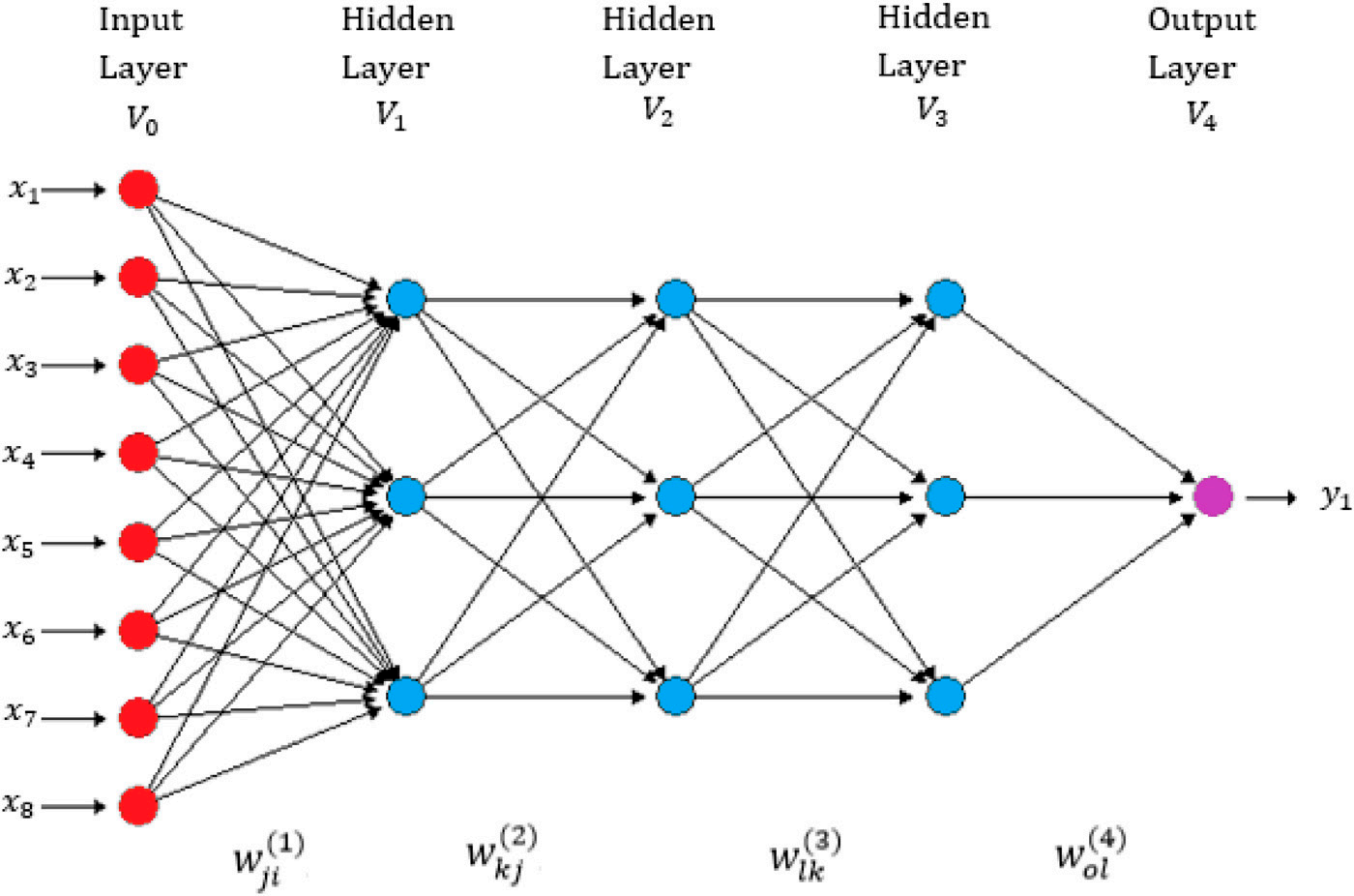
A feedforward deep neural network with one input layer, three hidden layers and one output layer (Montesinos-López, et al., 2021d). There are eight neurons in the input layer that correspond to the input information and four neurons in each of three hidden layers, with only one neuron in the output layer that corresponds to the count trait that will be predicted (Intercepts = biases not shown in this figure).

We implemented the most popular deep neural network architecture called densely connected networks (multilayer perceptron) (Chollet and Allaire, 2017). This network does not assume a specific structure in the input features. In general, the basic structure of a densely connected network consists of an input layer, one output layer (for uni-trait modeling) and multiple hidden layers between both layers. This type of neural network is also known as a feedforward deep neural network (See Figure 1). The implementation of this deep neural network is challenging because it requires many hyper-parameters, like number of units, number of layers, number of epochs, type of regularization method and type of activation function. Based on available literature, we used the rectified linear activation unit (ReLU) as the activation function in the hidden layers, the linear activation function in the output layer and the dropout type of regularization method for training the models (Chollet and Allaire, 2017).

The dataset was divided into training (80%) and testing (20%). Then each training set was divided into *inner-training* (80×0.8=64%) and *validation set* (80×0.2=16%) . With the *inner-training,* we trained the 8 resulting models (grid of eight values) by combining the following hyper-parameters: two neurons (1.5 × Number of independent variables of each dataset, 3 ×  Number of independent variables of each dataset), two values of hidden layers (with 1 and 4), two values of dropout (0.15 and 0.3), one learning rate equal to 0.001, and one value of epoch that was fixed at 1,000. From these eight combinations, we selected the best hyper-parameter combination in terms of prediction performance (with mean square error or prediction (MSE) in the *validation* set. Then, with the best hyper-parameter combination obtained from the *validation* set, a model was refitted with the whole information of the *training* (*inner-training* + *validation*) *set.* Then with this refitted model, predictions of the corresponding testing set were made. Finally, the average of the five folds in terms of MSE was reported as prediction performance of the conventional deep learning method (DL_M1). This model was evaluated with (GE) and without (NO GE) the genotype × environment interaction. When the GE was taken into consideration, the predictor contained the design matrix of environments $\left(X_{E}\right)$ , genotypes ( $X_{G}$ ; this matrix contains the raw design matrix of genotypes post multiplied by the Cholesky decomposition of the genomic relationship matrix) and the design matrix of the GE interaction ( $X_{GE};$ this matrix was built by combining matrices $X_{E}$ and $X_{G}$ ), that is, the predictor contained the following concatenated information: predictor=( $X_{E}$ , $X_{G}$ , $X_{GE}$ ). Conversely, when the GE was ignored (NO GE), the predictor only took into account the design matrices of $X_{E}$ and $X_{G},$ that is, predictor=( $X_{E}$ , $X_{G}$ ). However, it is important to point out that because deep neural networks apply more than one hidden layer, with many units (neurons) and with nonlinear transformations (activation functions) without explicitly giving the interaction term, $X_{GE}$ , they can capture complex interactions due to the way neurons interact with each other (See Figure 1).

### Calibration Method for Outputs of Deep Learning (DL_M2)

Next, we implemented the proposed new calibration method (DL-M2) for continuous outcomes. This involved eight steps, as follows. First, the data was divided into training (80%) and testing (20%) sets, as previously mentioned. Then the training data was divided into 1) an *inner-training* (80×0.8=64%) set and 2) a *validation* (80×0.2=16%) set. Following this, the *inner-training* was divided into *inner-inner-training* (64×0.8=51.2%) and *inner-validation* (64×0.2=12.8%) . With the *inner-inner-training* we trained the 8 resulting models (grid of eight values) of combining the following hyper-parameters: two neurons (1.5 × Number of independent variables of each data set, 3 ×  Number of independent variables of each data set), two values of hidden layers (with 1 and 4), two values of dropout (0.15 and 0.3), one learning rate equal to 0.001, and one value of epoch that was fixed at 1,000.

From these eight hyper-parameter combinations, we selected the best in terms of prediction performance in the *inner-validation* set. Then the best hyper-parameter combination obtained from the *inner-validation* set was refitted to a model with the information of the *inner-training* (*inner-inner-training* + *inner-validation*)*.* We then used the fitted model with the *inner-training* set to make predictions of the *validation* data set and for the testing data set. A linear model was then adjusted using the observed response variable of the *validation* set as the response variable and the predicted values (of the validation set) obtained in the fitting of the inner-training set for the *validation* data set as the independent variable. This step fits a linear model of the *observed validation* data with the *predicted validation* data previously obtained. Finally, we used this fitted linear model for making adjusted predictions of the testing set using only the predicted values of the testing set as input. This step provides adjusted predictions (calibrated) of the testing set and are the final predictions which are calibrated in this step. The steps in this process were repeated for each training-testing partition. In this case, there were five folds, and the average MSE of the five folds was reported as the prediction performance.

### Applying a Cross-Validation Strategy

To evaluate the predictive performance, we used a 5-fold cross-validation, with four folds used for training and one for testing. The average mean square error (MSE), was computed with the five folds, which was used to assess prediction performance in each data set under study. For deep learning models (DL_M1 and DL_M2), a 5-fold cross-validation was also implemented to select the best combination of hyper-parameters. For the conventional deep learning model (DL_M1), the 5-fold cross-validation was implemented with a training set that was divided into inner-training and validation, while for the proposed calibration method (DL_M2), the 5-fold cross-validation was implemented with the inner-training set that was divided into inner-inner-training and inner-validation. This strategy of cross validation mimics real applications where some lines are missing in some environment’s, but are present in at least another environment. This means that our approach does not mimic scenarios where we use previous generation to predict next generations as training. On the other hand, as pointed out by one reviewer, other metrics can be used for evaluating the prediction accuracy like the Person´s correlation, even though in this application only the MSE was used. Furthermore, in this case since we have available phenotypes (because a 5-fold cross-validation approach was used), it was not necessary to compare predictions with parent averages from early generations.

## Results

### Dataset 1 (Maize Data set)

**FIGURE 2:**
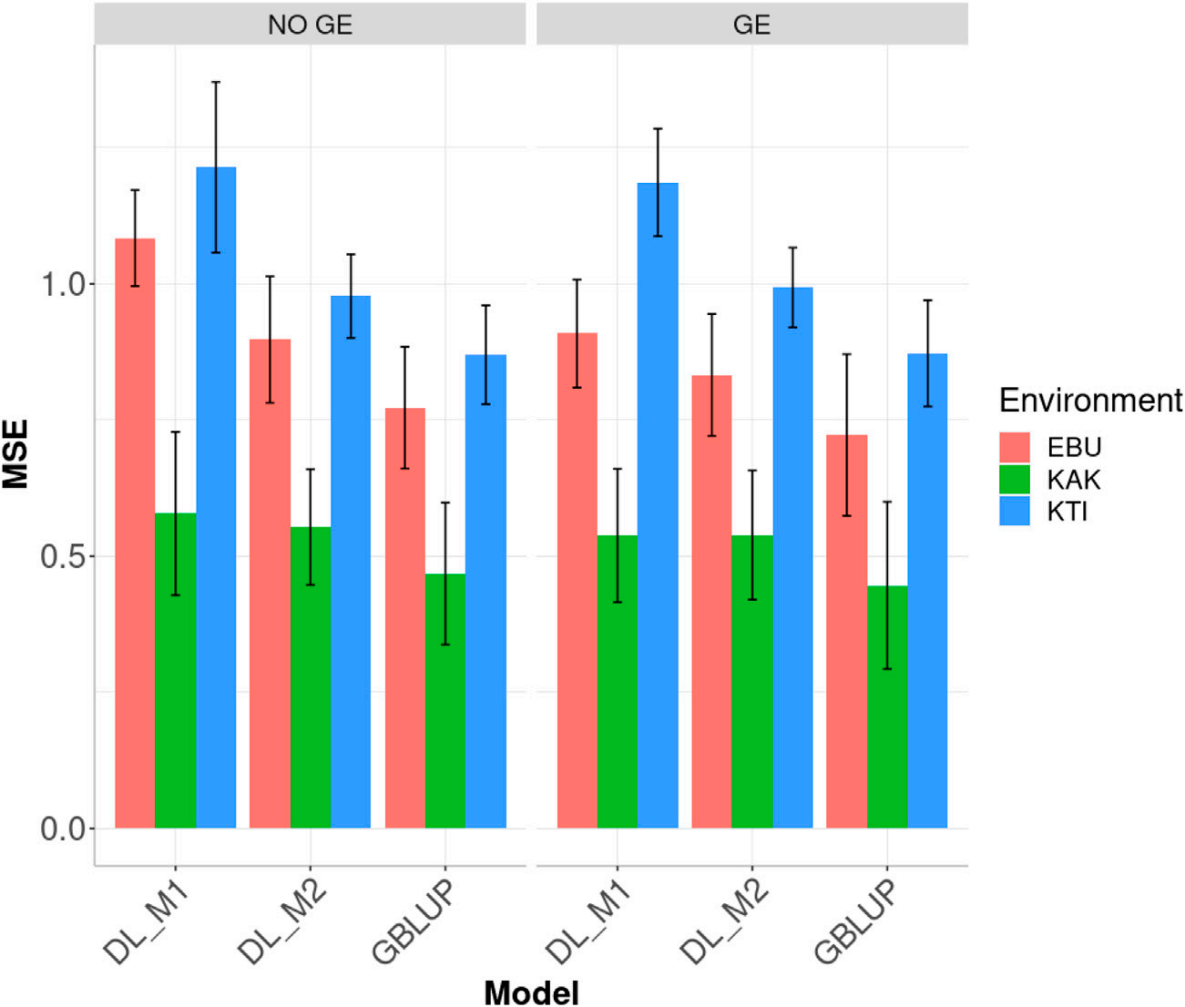
Dataset 1. Prediction performance in terms of mean square error (MSE) for each environment of the maize dataset under models: DL_M1, DL_M2 and GBLUP. No GE means that the model ignores the genotype ×environment interaction. GE means that the model takes the genotype × environment interaction into account.

Analysis of Dataset 1 showed that the best prediction performance when including genotype × environment (GE) interaction was observed using GBLUP (Figure 2). Across the sites, the best predictions were observed under environment KAK while the worst were observed under environment KTI. When comparing the conventional deep learning method (DL_M1) with our proposed method for calibrating deep learning models (DL_M2), we observed that the proposed calibration method improved the genome-enabled prediction performance based on MSE. Ignoring the GE interaction term in environments EBU, KAK and KTI, DL_M2 reduced the MSE with regard to DL_M1 by 17.188, 4.273 and 19.469%, respectively. However, the DL_M2 prediction performance in terms of MSE was worse than the GBLUP method, which outperformed DL_M2 at all sites by 16.179% (EBU), 18.298% (KAK) and 12.370% (KTI).

When the GE interaction was taken into account, the DL_M2 method reduced the MSE compared to DL_M1 by 8.354, 1.487 and 16.258% in environments EBU, KAK and KTI, respectively. However, although the improvement of DL_M2 over DL_1 is significant, the GBLUP method still outperformed DL_M2 method by 15.268, 20.753 and 13.848% in environments EBU, KAK and KTI, respectively.

**FIGURE 3:**
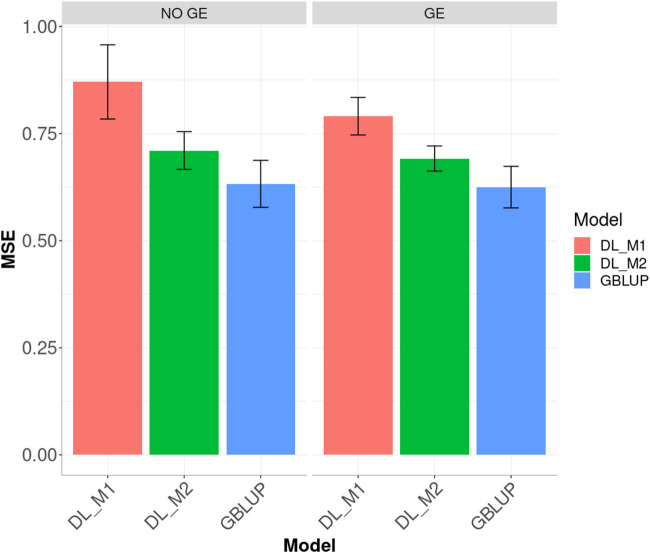
Dataset 1. Prediction performance in terms of mean square error (MSE) across environments for the maize data set under models: DL_M1, DL_M2 and GBLUP. No GE means that the model ignores the genotype ×environment interaction. GE means that the model takes the genotype × environment interaction into account.

Finally, across environments (Figure 3), the best prediction performance was observed from the GBLUP and the worst from the DL_M1 method. GBLUP outperformed DL_M2 by 12.296% and DL_M1 by 37.616%, whilst DL_M2 outperformed DL_M1 by 18.399%. Accounting for the GE interaction, GBLUP outperformed both the DL_M1 and DL_M2 by 26.491 and 10.686%, respectively. With GE interaction, DL_M2 reduced the MSE compared with the DL_M1 by 12.495%.

### Dataset 2 (Groundnut Dataset)

**FIGURE 4:**
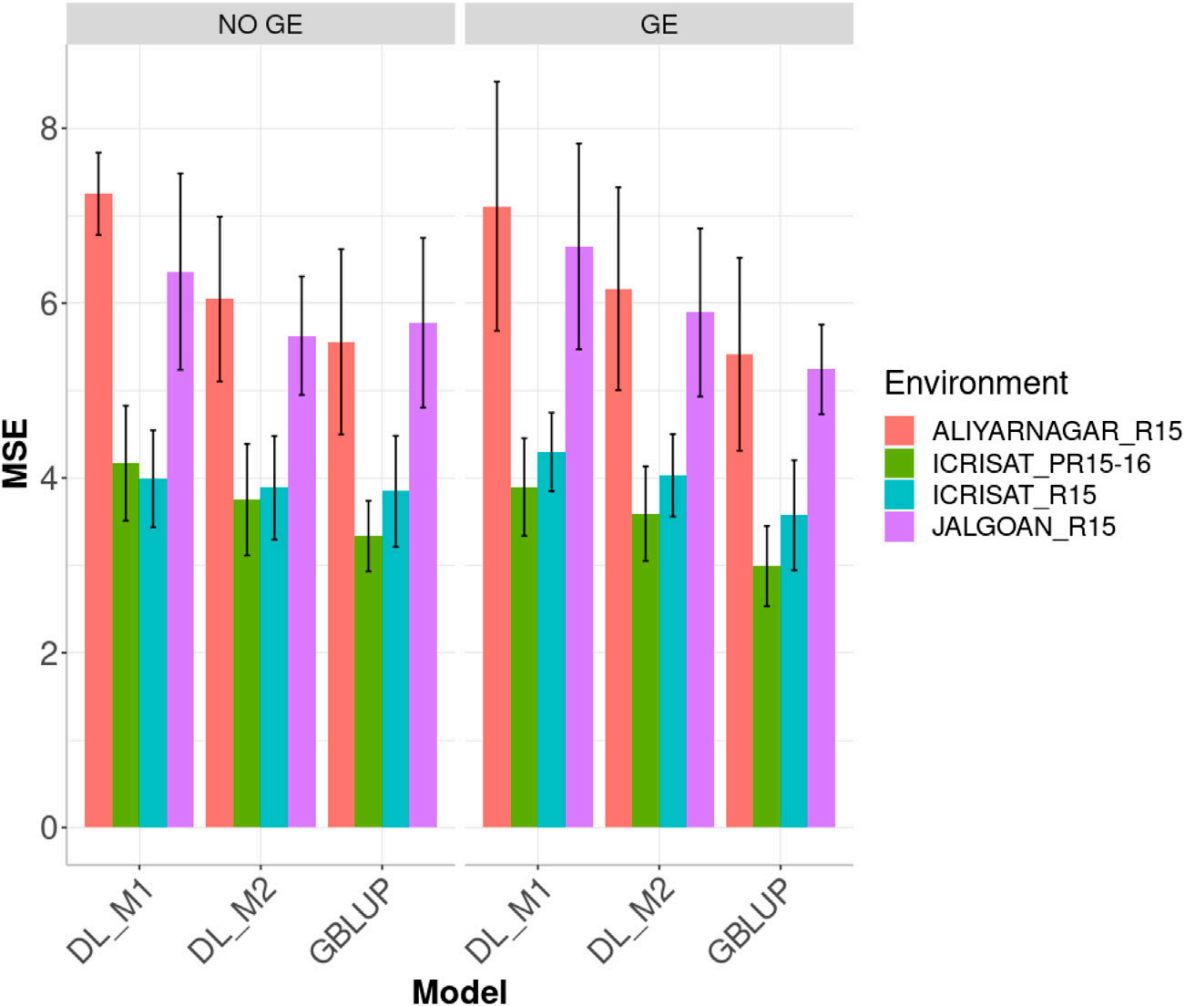
Dataset 2. Prediction performance in terms of mean square error (MSE) for each environment of the groundnut dataset under models: DL_M1, DL_M2 and GBLUP. No GE means that the model ignores the genotype ×environment interaction. GE means that the model takes the genotype × environment interaction into account.

Figure 4 displays the genome-enabled prediction performance (MSE) including (or not) the GE interaction under the GBLUP, DL_M1 and DL_M2 methods in the four environments. The proposed method of calibration (DL_M2) improved the prediction performance of conventional deep learning method (DL_M1). When ignoring the GE interaction term, the DL_M2 method reduced the MSE compared with DL_M1 by 16.591, 10.004, 2.538 and 11.514% in environments ALIYARNAGAR_R15, ICRISAT_PR15-16, ICRISAT_R15 and JALGOAN_R15, respectively. The GBLUP method outperformed the DL_M2 method in three out of the four environments by 8.80% (ALIYARNAGAR_R15), 12.519% (ICRISAT_PR15-16) and 1.096% (ICRISAT_R15). The worst predictions were observed under environments ALIYARNAGAR_R15 and JALGOAN_R15, while the best were observed under environments ICRISAT_PR15-16 and ICRISAT_R15.

**FIGURE 5:**
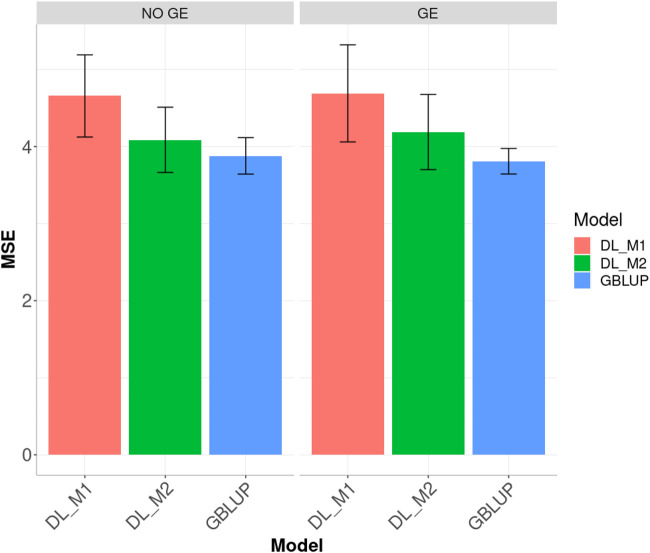
Dataset 2. Prediction performance in terms of mean square error (MSE) across environment of the Groundnut dataset under models: DL_M1, DL_M2 and GBLUP. No GE means that the model ignores the genotype × environment interaction. GE means that the model takes the genotype × environment interaction into account.

Considering GE interaction, the DL_M2 method improved the prediction performance compared with the DL_M1 method in environments ALIYARNAGAR_R15, ICRISAT_PR15-16, ICRISAT_R15 and JALGOAN_R15 by 13.256, 7.766, 6.238 and 11.368%, respectively. The GBLUP method overcame the DL_M2 method by 13.864, 20.078, 12.763 and 12.423% in environments ALIYARNAGAR_R15, ICRISAT_PR15-16, ICRISAT_R15 and JALGOAN_R15, respectively (Figure 5).

When ignoring the GE interaction across environments (Figure 5), the worst predictions were observed under the DL_M1 method and the best under the GBLUP method. The GBLUP outperformed the DL_M2 method by 5.535%, while the DL_M2 outperformed the DL_M1 by 12.229%; the GBLUP was also better than the DL_M1 by 20.045%. When including GE interaction, results showed that DL_M1 was the worst method and the GBLUP was the best, but now the GBLUP outperformed the DL_M1 and DL_M2 methods by 23.116 and 9.929%, respectively. However, for this dataset, DL_M2 outperformed the DL_M1 by 10.711%.

### Dataset 3 (Chickpea)

**FIGURE 6:**
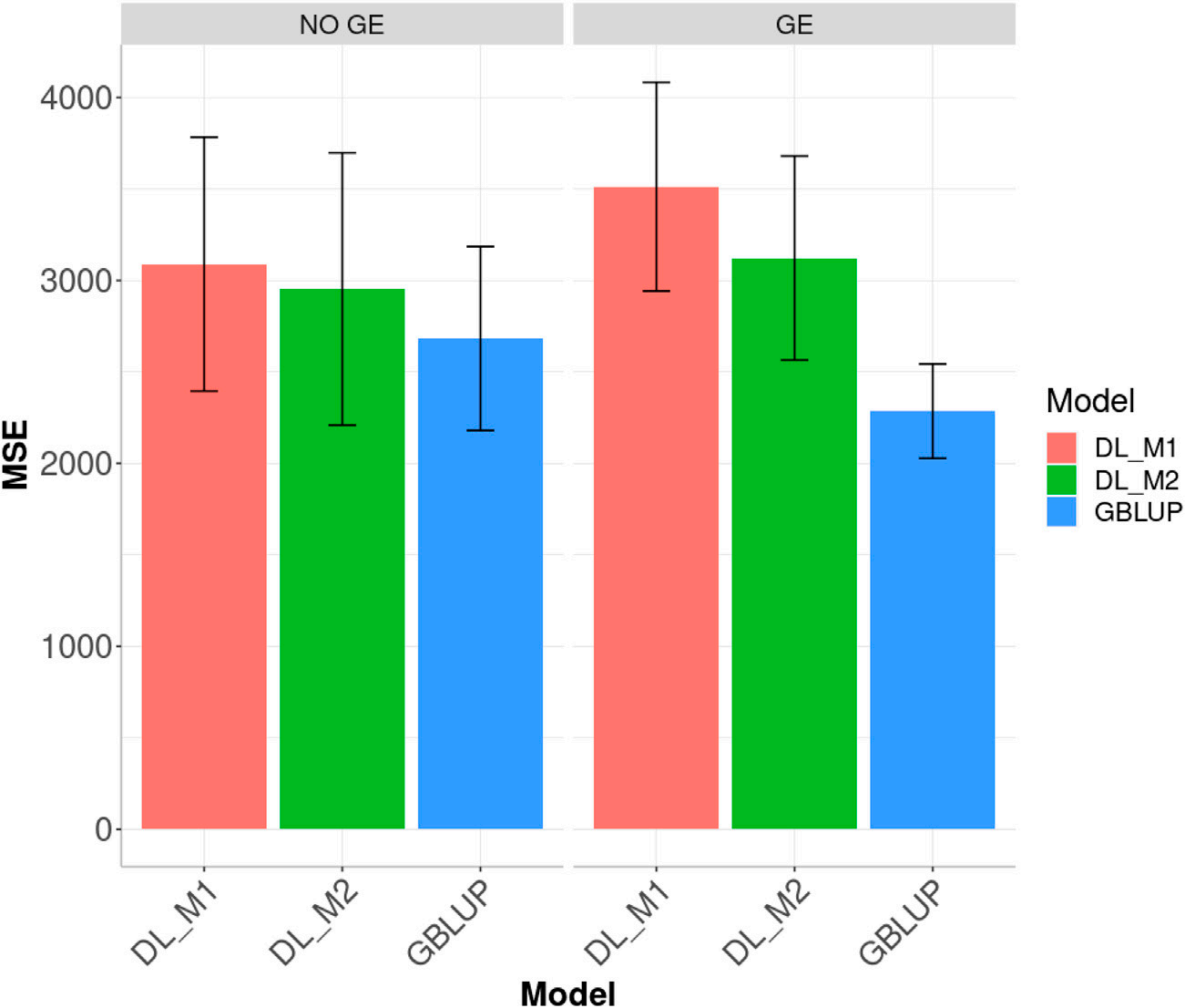
Dataset 3. Prediction performance in terms of mean square error (MSE) across environments for the chickpea dataset under models: DL_M1, DL_M2 and GBLUP. No GE means that the model ignores the genotype × environment interaction. GE means that the model takes the genotype × environment interaction into account.

Ignoring GE in the across environments case, Figure 6 indicates that the best predictions were observed under the GBLUP method and the worst under the DL_M1 method. Furthermore, the GBLUP outperformed the DL_M2 method by 10.082%, while the DL_M2 outperformed the DL_M1 by 4.402% and the GBLUP outperformed the DL_M1 by 15.152%. Considering the GE interaction, the GBLUP was the best method and the DL_M1 the worst, where the GBLUP outperformed the DL_M1 and DL_M2 by 53.679 and 36.616%, respectively. Results show that compared with the DL_M1 method, the DL_M2 reduced the MSE by 11.102%.

### Dataset 4 [Elite Wheat Yield Trial (EYT) Year 2016–2017]

**FIGURE 7:**
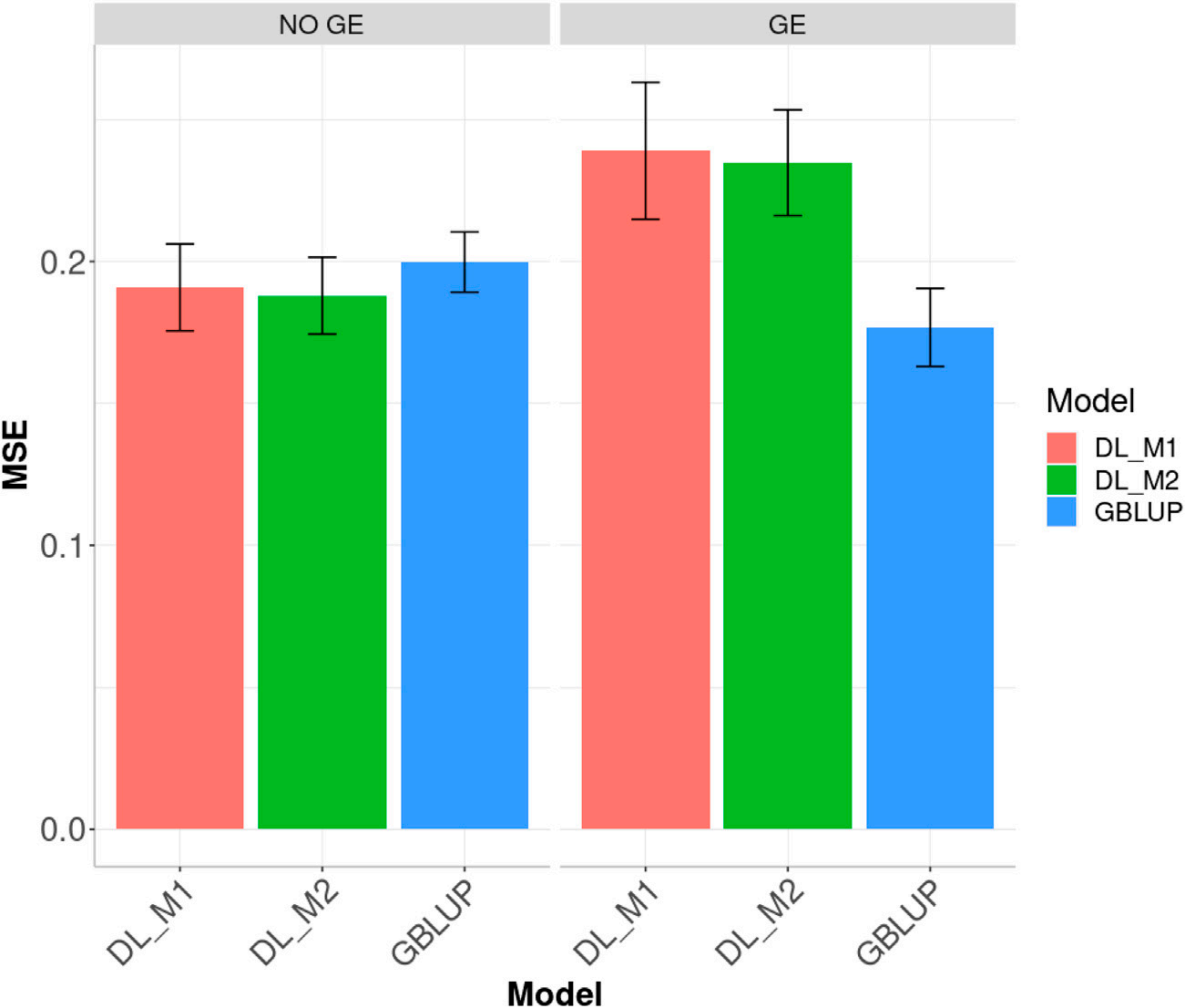
Dataset 4. Prediction performance in terms of mean square error (MSE) across environments for the elite wheat yield trial (EYT) year 2016–2017 dataset under models: DL_M1, DL_M2 and GBLUP. No GE means that the model ignores the genotype × environment interaction. GE means that the model takes the genotype × environment interaction into account.

Results for across environments are displayed in Figure 7. When no GE was included, the best predictions were observed under the DL_M2 method and the worst under the GBLUP method; the DL_M2 outperformed the GBLUP method by 5.906%, while the DL_M2 outperformed the DL_M1 by 1.519% and the DL_M1 outperformed the GBLUP by 4.454%. When considering the GE interaction, the GBLUP was the best and the DL_M1 the worst, where GBLUP outperformed the DL_M1 and DL_M2 by 35.18 and 32.805%, respectively. Compared with the DL_ M1, the DL_M2 reduced the MSE by 1.757%.

## Discussion

Genomic selection helps save significant resources for the early selection of candidate genotypes because instead of phenotyping and genotyping all the candidate lines, only a sample of them are phenotyped and genotyped. For the remaining individuals that were only genotyped, genome-enabled predictions of the phenotypic values are performed. This means that the accuracy of GS is linked to the quality of the predictions, and the better the predictions, the more accurate the GS methodology. Thus, continuing research to improve the quality of the predictions using GS is of paramount importance. For this reason, this research proposed a simple and novel calibration method to improve the predictions resulting from deep learning methods.

The proposed method was evaluated in four datasets and we found that in three out of the four datasets, the proposed calibration method improved the predictions over conventional deep learning methods. The increase in prediction performance in these four datasets was between 1.519 and 18.39% across environments, which empirically reflects that the proposed calibration method (DL_M2) is quite efficient for improving the prediction power of deep learning models. However, it is important to point out that we did not find that the proposed calibration method outperformed the predictions of the GBLUP method, one of the most popular genomic prediction models. In fact, the GBLUP method outperformed the proposed calibration method (DL_M2) across environments between 5.535 and 36.616% in the four data sets.

However, taking into account the standard errors in most of the scenarios under study no statistical differences were observed between the proposed DL_M2 and the GBLUP method. Two reasonable explanations as to why the GBLUP method outperformed the proposed deep learning method even with the proposed calibration method could be that the four data sets are: 1) small and, as pointed out above, the deep learning methods are data hungry, and 2) they do not have complex nonlinear patterns. It is also important to highlight that our results only used markers (not pedigree) information, whereas some researchers have reported similar results using both pedigree and markers (Ankamah-Yeboah et al., 2020; Calleja-Rodriguez, et al., 2020). Furthermore, the training process with the two deep learning methods (DL_M1 and DL_M2) was considerable slower than the conventional GBLUP method.

The proposed method is attractive for four reasons: 1) its implementation is straightforward, 2) it is a post-processing method, 3) it helps increase the prediction performance of deep learning methods and 4) even with a small grid for the tuning process of the DL model, the proposed calibration method will provide reasonable predictions. We observed that the proposed DL method works better with smaller data sets, which is of paramount importance because the lower the data set, the harder the training process of deep learning methods become, since it is well documented that deep learning methods are data hungry (Chollet and Allaire, 2017; Chollet, 2018). In deep learning methods, the prediction accuracy is strongly influenced by the sample size, the heritability, the genetic architecture of the trait of interest, the genome structure of the species under study (Daetwyler et al., 2010; Schopp et al., 2017) and the mating design and family structure of the training set (Hickey et al., 2014). The relatedness between the training and testing sets also plays an important role (Habier et al., 2007; Saatchi et al., 2011; Clark et al., 2012; Lorenz and Nice 2017).

Note that the results presented in this study are not completely definitive, as more empirical evidence is required to be able to claim that the proposed method really helps improve the prediction performance of deep learning methods. The current results are attractive since we observed that the proposed calibration method (DL_M2) helped to more efficiently train deep learning models with an increase in prediction accuracy over the conventional DL methods (DL_M1) that is not negligible. Although the conventional GBLUP method behaves as the best model for the medium to large size data sets included in this study, there are cases where the DL_M1 and the DL_M2 overcame GBLUP genome-enabled prediction, as in the case of data set 4 (wheat data set) and provide similar results to those obtained by Montesinos-Lopez et al. (2019a,b).

DL methods should be used over conventional linear models (like the GBLUP) when it is suspected that the data contain nonlinear patterns. In this vein, one important advantage of DL over conventional methods for genome-enabled prediction is that the whole genetic merit, including all non-additive effects, can potentially be predicted without the need to partition all effects (Zingaretti et al., 2020). It should also be noted that DL consists of a number of layers of neurons and is a hierarchical information extraction process, which is exemplified by the classifications of objects by DL with images (Lee et al., 2009; Chollet, 2018). In the first layer, neurons detect simple and basic features of objects; in the intermediate layers, they detect parts of objects (Chollet and Allaire, 2017; Chollet, 2018); and in the top layers, they code for objects. For this reason, as pointed out in, DL offers many areas of opportunities that should be explored in order to take the full advantage of this technology. Some of these areas of opportunities are:

1) modifying, adapting, or inventing new DL architectures, activation functions, and tuning strategies for the specific context of GS;
2) adapting, improving, and developing more user-friendly software for DL applications in GS;
3) performing greater benchmarking studies to compare the prediction performance of existing DL methods to those that are the standard genome-enabled predictions in GS;
4) exploring transfer learning for GS. The goal of transfer learning is to use the knowledge learned from one specific set of environments to ease the learning tasks in another different but similar environment;
5) exploring how to use reinforcement learning in the context of GS;
6) exploring deep generative models (generative adversarial networks (GANs) and variational auto-encoder (VAE) methods to generate new inputs (fictitious markers or independent variables) that are indistinguishable from the original training set;
7) training or retraining breeders and people involved in genomic prediction in these new frameworks for DL, as exemplified by Keras (Chollet and Allaire, 2017);
8) exploring the deep compression methods in GS to reduce the computation and storage required by neural networks;
9) increasing our efforts for data sharing in platforms to create large data sets for each species containing not only phenotypic and markers data, but also environmental information and other omics data (Montesinos-López et al., 2021a,c,d);
10) taking advantage of DL tools to include in the predictor imaging information that is being collected in plants and to also measure using computer vision tools phenotypic properties that are fast, non-invasive and low-cost (Fahlgren et al., 2015).

Nevertheless, researchers and practitioners must be aware that DL is not always the right method, and for this reason, we need to be open to trying other models. As pointed out in Montesinos-López et al. (2021a), there is still not enough empirical evidence that deep learning methods outperform conventional genomic prediction models or that DL methods are more computationally demanding. However, we need to be conscious that deep learning is just starting to be used in genetics and plant breeding and is not well researched, especially for its optimal implementation in this area of research (Varshney R. K. et al., 2021). In this study, we used a feed-forward deep neural network also known as multilayer perceptron neural network and, for this reason, our results are limited to this type of architectures (topologies). More empirical evaluations are needed to corroborate that the proposed method is also efficient for other deep learning architectures.

## Conclusion

In this paper we proposed a simple calibration method for outputs from deep learning methods with continuous response variables. We found that the proposed calibration methods help to significantly improve prediction accuracy obtained from deep learning methods, and even greater improvement in smaller data sets. The proposed DL method contributes to the training process in the context of small data sets. However, we suggest performing more empirical evaluations to accumulate more evidence of the utility of the proposed calibration method. Another advantage of the proposed calibration method is that it is a post-processing method that is very simple to implement, as it involves only a few and simple steps. In general, results demonstrated that no unique model/method exists for producing the most accurate genome-enabled predictions.

## Data Availability

Publicly available datasets were analyzed in this study. This data can be found here: https://github.com/brandon-mosqueda/dlc-datasets.
